# EH Domain-Containing 2 Deficiency Restricts Adipose Tissue Expansion and Impairs Lipolysis in Primary Inguinal Adipocytes

**DOI:** 10.3389/fphys.2021.740666

**Published:** 2021-09-24

**Authors:** Claes Fryklund, Björn Morén, Shrenika Shah, Mario Grossi, Eva Degerman, Claudia Matthaeus, Karin G. Stenkula

**Affiliations:** ^1^Department of Experimental Medical Science, Lund University, Lund, Sweden; ^2^School of Biomedical, Nutritional and Sport Sciences, Newcastle University, Newcastle upon Tyne, United Kingdom; ^3^National Heart, Lung and Blood Institute, NIH, Bethesda, MD, United States

**Keywords:** adipocytes, EH domain-containing 2, caveolae, fatty acids, adipose tissue

## Abstract

Lipid uptake can be facilitated *via* caveolae, specific plasma membrane invaginations abundantly expressed in adipocytes. The dynamin-related protein EH domain-containing 2 (EHD2) stabilizes caveolae at the cell surface. Here, we have examined the importance of EHD2 for lipid handling using primary adipocytes isolated from EHD2 knockout (*Ehd2^−/−^*) C57BL6/N mice. Following high-fat diet (HFD) feeding, we found a clear impairment of epididymal, but not inguinal, adipose tissue expansion in *Ehd2^−/−^* compared with *Ehd2^+/+^* (WT) mice. Cell size distribution analysis revealed that *Ehd2^−/−^* mice had a lower proportion of small adipocytes, and an accumulation of medium-sized adipocytes in both epididymal and inguinal adipose tissue. Further, PPARγ activity, FABP4 and caveolin-1 expression were decreased in adipocytes isolated from *Ehd2^−/−^* mice. Inguinal adipocytes isolated from *Ehd2^−/−^* mice displayed reduced lipolysis in response to beta adrenergic receptor agonist, which was associated with reduced phosphorylation of perilipin-1 and hormone sensitive lipase (HSL). This impairment could not be rescued using a cAMP analog, indicating that impaired lipolysis in *Ehd2^−/−^* primary adipocytes likely occurs at the level of, or downstream of, protein kinase A (PKA). Altogether, these findings pinpoint the importance of EHD2 for maintained intracellular lipid metabolism, and emphasize differences in mechanisms regulating lipid handling in various adipose-tissue depots.

## Introduction

There is a global rise in obesity, and it is estimated that 38% of the population will have overweight by year 2030. During excess calorie intake, both hypertrophy and hyperplasia of adipocytes occur, ultimately causing adipose tissue expansion (Faust et al., 1978; Li et al., 2016). Adipocyte size predicts development of type 2 diabetes (Weyer et al., 2000; Acosta et al., 2016) and sustained overconsumption of calories leads to an accumulation of hypertrophic adipocytes that are less responsive to insulin (Salans et al., 1968; Salans and Dougherty, 1971; Smith, 1972; Franck et al., 2007). Impaired differentiation (Grunberg et al., 2014; Acosta et al., 2016) and a limited ability of adipocytes to expand further (McLaughlin et al., 2007) could contribute to adipose tissue dysfunction. Notably, the adipogenic potential varies in different adipose tissue depots (Wang et al., 2013; Jeffery et al., 2015). For example, a restricted capacity to increase the subcutaneous adipose tissue is hypothesized to favor expansion of visceral adipose tissue and underlie progression toward metabolic disorders (Wajchenberg, 2000; McLaughlin et al., 2011; Shulman, 2014).

Caveolae are specific invaginations of the plasma membrane (Palade, 1953; Yamada, 1955). These specialized domains orchestrate lipid transport (Razani et al., 2002; Pohl et al., 2005; Meshulam et al., 2006; Liu et al., 2008), and *via* interaction with the caveolar core protein caveolin, caveolae scaffold various signaling molecules, including endothelial nitric oxide synthase (eNOS; Garcia-Cardena et al., 1996), tyrosine kinase receptors (Gustavsson et al., 1999), and G-protein coupled receptors (Schwencke et al., 1999; Insel et al., 2005). Ablation or mutation of caveolins, or caveolae depletion, lead to impaired insulin signaling in adipose tissue (Gustavsson et al., 1999; Cohen et al., 2003) and lipodystrophy in rodent models (Razani et al., 2002; Liu et al., 2008) and human (Cao et al., 2008; Kim et al., 2008). Indeed, caveolin-1 deficient mice were resistant to diet-induced obesity (Razani et al., 2002). EH domain-containing 2 (EHD2) is a dynamin-related ATPase (Daumke et al., 2007) that localizes at the neck of caveolae, where it is proposed to regulate the stability of caveolae at the plasma membrane (Moren et al., 2012; Stoeber et al., 2012; Ludwig et al., 2013). Further studies have addressed caveolae as a membrane reservoir, where EHD2 serves as a mechanosensor, and *via* translocation to the nuclei connects caveolae with transcriptional activity (Torrino et al., 2018). Recently, we identified *Ehd2* as one of the most highly upregulated genes in adipose tissue after short-term overfeeding in mice (Hansson et al., 2018). In a follow-up study, we demonstrated that EHD2 associates and drives the formation of cell surface-proximal lipid droplets in adipocytes, sustains *in vitro* adipogenesis and enhances lipolytic activity (Moren et al., 2019). In line with our results, EHD2 was confirmed to affect lipid metabolism in *Ehd2^−/−^* mice (Matthaeus et al., 2020). In that study, EHD2 deficiency led to caveolae detachment and increased fatty acid uptake, reinforcing the role of caveolae for lipid transport.

Even though caveolae are undoubtedly involved in lipid metabolism, the specific mechanisms are currently not understood. While recent studies have addressed EHD2 to play a role in lipid handling, it is unclear how EHD2 deficiency affects these events in primary adipocytes. Furthermore, it is unknown if EHD2 influences gene transcription activity that is essential to sustain adipose tissue expansion. To address this, we have characterized adipose cell size distribution and lipolysis in primary adipocytes isolated from *Ehd2^−/−^* mice challenged with high-fat diet (HFD) feeding. We demonstrate that EHD2 is necessary for both adipose tissue expansion and for maintenance of lipolysis in primary adipocytes.

## Materials and Methods

### Material

Adipose triglyceride lipase (ATGL; #2138), caveolin-1 (#3267S), FABP4 (#3544S), hormone sensitive lipase (HSL) pS563 (#4139), CREB pS133 (#9191S) and pPKA motif specific [P-RXX(S/T); #9624S] antibodies were from Cell Signaling Technologies (Danvers, United States). Heat shock protein (HSP) 90 (#610418) antibody was from BD Transduction Laboratories (Franklin Lakes, United States). Perilipin-1 total (#4854) and pS522 (#4856) antibodies from Vala Science (San Diego, United States). Antibody against EHD2 (#154784) was from Abcam (Cambridge, United Kingdom). Total HSL antibody was kindly provided by Cecilia Holm (Lund University). Fluorescence-conjugated (Alexa Fluor) secondary antibodies, Bodipy 493/503 (D3922), and MitoTracker RED CMXRos (M7512) were purchased from Molecular Probe (Waltham, United States). Rosiglitazone (R2408) was from Sigma-Aldrich (St. Louis, United States). The PPRE-x3-TK-Luc reporter plasmid was a kind gift from Bruce Spiegelman (Addgene plasmid #1015) and Renilla Luciferase Control Reporter (pRLnull; #E227A) from Promega (Madison, United States). Reagent for determination of glycerol content (F6428) and dibutyryl-cAMP (DcAMP; #D0627) were from Sigma-Aldrich (St. Louis, United States). PDE3 inhibitor OPC3911 and PDE4 inhibitor Rolipram were from Otsuka Pharmaceuticals (Tokyo, Japan) and PDE3B antibody was produced in-house.

### Animals

C57BL6/N mice with global deletion of exon 3 of the Ehd2 gene (*Ehd2^−/−^*) as described (Matthaeus et al., 2020) were kindly provided by Oliver Daumke (MDC, Germany). Genotyping was carried out to confirm deletion of the *Ehd2* gene. *Ehd2^−/−^* and *Ehd2^+/+^* C57BL6/N mice (the latter used as WT control) were bred in parallel to generate sufficient number of animals for cellular analysis. Animals were on a 12h light cycle with non-restricted food and water. Mice were fed either chow or HFD (D12492 60 E% fat content; Research Diets, New Brunswick, United States).

### Ethics Statement

All animal procedures were approved by the Malmö/Lund Committee for Animal Experiment Ethics, Lund, Sweden, and were carried out in accordance with the relevant guidelines and regulations.

### Adipocyte Isolation

Primary adipocytes were isolated from different adipose tissue depots using previously established protocol (Rodbell, 1964). Isolated, primary adipocytes were suspended 10% (v/v) in Krebs Ringer Bicarbonate HEPES (KRBH) buffer, pH 7.4, containing 200nM adenosine and 3% (w/v) bovine serum albumin (BSA).

### Western Blot

Following incubations, cells were washed with KRH medium without BSA and subsequently lysed in a buffer containing 50mM Tris/HCl pH 7.5, 1mM EGTA, 1mM EDTA, 0.27M sucrose, 1% NP-40, and complete protease- and phosphatase inhibitor cocktail (Roche, Basel, Switzerland). Lysates were centrifuged for 10min at 13,000×*g* and protein concentrations were determined using the Bradford method. Samples were subjected to polyacrylamide gel electrophoresis and electro-transfer to nitrocellulose membranes. Membranes were blocked with non-fat dry milk [10% (w/v)] and probed with the indicated antibodies. Detection was performed using horseradish peroxidase conjugated secondary antibodies and enhanced chemiluminescence reagent. The signal was visualized using a BioRad Image camera (Biorad, Hercules, United States).

### Plasmid Transfection and Luciferase Activity Assays

Adipocytes were transfected as previously described (Lizunov et al., 2013). In short, isolated adipose cells were suspended [40% (v/v)] in DMEM supplemented with Gentamicin (20 µg/ml) and 200nM phenylisopropyladenosine (PIA). Cells were electroporated using a square-wave pulse; 400V, 12ms, one pulse (Harvard Apparatus, Holliston, United States) with PPRE reporter and pRLnull (normalization) plasmids (10:1). After electroporation, the cells were transferred into DMEM with Gentamicin (20μg/ml), PIA and [3.5% (w/v)] BSA and cultured for 24h at 37°C in 5% CO_2_. Thereafter, the cells were lysed in Promega passive lysis buffer, and the lysates centrifuged at 10,000×*g* for 10min at 4°C to separate the protein lysate (infranatant) from the fat. Luciferase activity was measured in a Glomax luminometer (Promega) using the Dual Luciferase Reporter system (Promega), where data were normalized to Renilla reporter (pRLnull).

### Fluorescence Microscopy

Imaging was performed using a Nikon A1 plus confocal microscope with a 60× Apo DIC oil immersion objective with a NA of 1.40 (Nikon Instruments) and appropriate filter sets. Images were acquired with NIS-Elements, version: 4.50.02 (Laboratory Imaging). Isolated cells were fixed using 4% paraformaldehyde (PFA) and labeled with antibodies in a buffer containing 1% BSA, 1% goat serum, and 0.05% saponin 1–2h. For neutral lipid staining, cells were stained with Bodipy 493/503 and for mitochondria we used Mitotracker. For imaging of primary adipocytes, we used previously described protocol (Wasserstrom et al., 2018).

### Electron Microscopy

For standard trans-electron microscopy, adipose tissue was fixed overnight using 1% PFA and 3% glutaraldehyde, followed by dehydration using methanol series (20, 40, 60, 80, and 100%) 30min each, de-lipidated using dichloromethane for 1h, and then rehydrated with reverse methanol series. Methanol was diluted in KRBH without BSA. The samples were fixed using osmium-tannic acid-osmium (OTO) for 1h each, washed in KRBH without BSA, dehydrated using ethanol series and embedded in EPON.

### Lipolysis

Lipolysis was determined by measuring glycerol release, as described previously (Hansson et al., 2016). In short, cells [10% (v/v) cell suspension] were treated with or without isoprenaline (ISO; 10nM), or a combination of insulin (1nM) and ISO (10nM) for 30min. Cell medium was subsequently removed for enzymatic determination of the glycerol content. In short, 100μl free glycerol reagent was added to 30μl sample. Absorbance at 540nm was measured after incubating for ~15min at room temperature. Each sample was analyzed in duplicate. In a separate set of experiments, cells were pre-incubated (10min) with OPC3911 (10μM), Rolipram (10μM) or DMSO 0.1% (control), followed by 30min stimulation with or without ISO (10nM) or a combination of insulin (1nM) and ISO (10nM). For experiments where DcAMP was used, cells were pre-incubated for 10min and then treated with or without ISO (10 or 1,000nM) or DcAMP (100 or 1,000μM) for 30min. Glycerol release was measured as stated above.

### RT-qPCR

Total RNA was extracted using miRNeasy mini kit (Qiagen #74104). RNA purity and concentration were assessed using a Nanodrop spectrophotometer (Thermo Scientific). PCR-reactions were performed using the Quantifast SYBR Green RT-PCR kit (Qiagen #204156) and Quantitect primer assays for *18S*, *Adrb3*, *Ehd2*, and *Plin1*. Primer sequences are considered proprietary information by Qiagen. mRNA expression levels were measured using a StepOnePlus real-time thermal cycler (Applied Biosystems Waltham, United States) and quantitated using the ΔΔC_T_ method as described by Livak and Schmittgen (2001). 18S rRNA was used for normalization throughout.

### Cell Size Distribution

Adipose tissue samples were obtained from epididymal (EPI) and ING adipose depots. The adipose cell-size distributions were obtained using a Beckman-Coulter counter after osmium fixation as described previously (Li et al., 2016).

### Liver Triglyceride Content

To determine the liver triglyceride concentration, ~100mg frozen liver sample was homogenized using a motorized homogenizer in buffer containing 5% (w/v) NP-40. The homogenized samples were heated at 90°C for 3min, cooled down for 15min in room temperature, reheated at 90°C for 3min, and then centrifuged at maximum speed for 2min at 20°C. The supernatant was collected and placed in a 96-well plate with triglyceride reagent (Thermo Scientific) for triglyceride concentration determination.

### Blood Analyses

Terminal serum samples were collected and blood glucose levels measured using OnetouchUltra2 (Lifescan, Milpitas, United States), and insulin levels assayed in serum using ELISA from Mercodia (Uppsala, Sweden). Serum concentration of free fatty acid (FFA) was measured using FFA assay kit (#ab65341) from Abcam (Cambridge, United Kingdom).

### Oil Red-O Staining

Cut frozen sections (5μm) were fixed with 4% PFA and incubated for 10min at room temperature. Excess of 4% PFA was removed by three rinses in PBS. Subsequently, the sections were rinsed with 60% isopropanol and then stained with freshly and filtrated oil Red-O working solution [30ml of 0.5% ORO (O0625, Sigma; Saint Louis, United States) stock solution+20ml distilled water] for 20min at room temperature. After the sections were rinsed with 60% isopropanol and then washed with distilled water and covered with a coverslip using permanent aqueous mounting medium (BUF058A, Biorad). Images were captured using an Olympus BX60 microscope.

### Statistical Analysis

Analysis was performed by unpaired two-tailed Student’s *t*-test or one-way ANOVA, Tukey’s *post hoc* test using GraphPad Prism 6 (Graphpad Software Inc.) software. Significance was determined according to ^*^p<0.05, ^**^p<0.01, and ^***^p<0.001.

### Data Availability Statement

The data that support the findings of this study are available from the corresponding author upon reasonable request.

## Results

### Decreased Amount of Visceral Adipose Tissue in *Ehd2^−/−^* Mice

**Figure 1:**
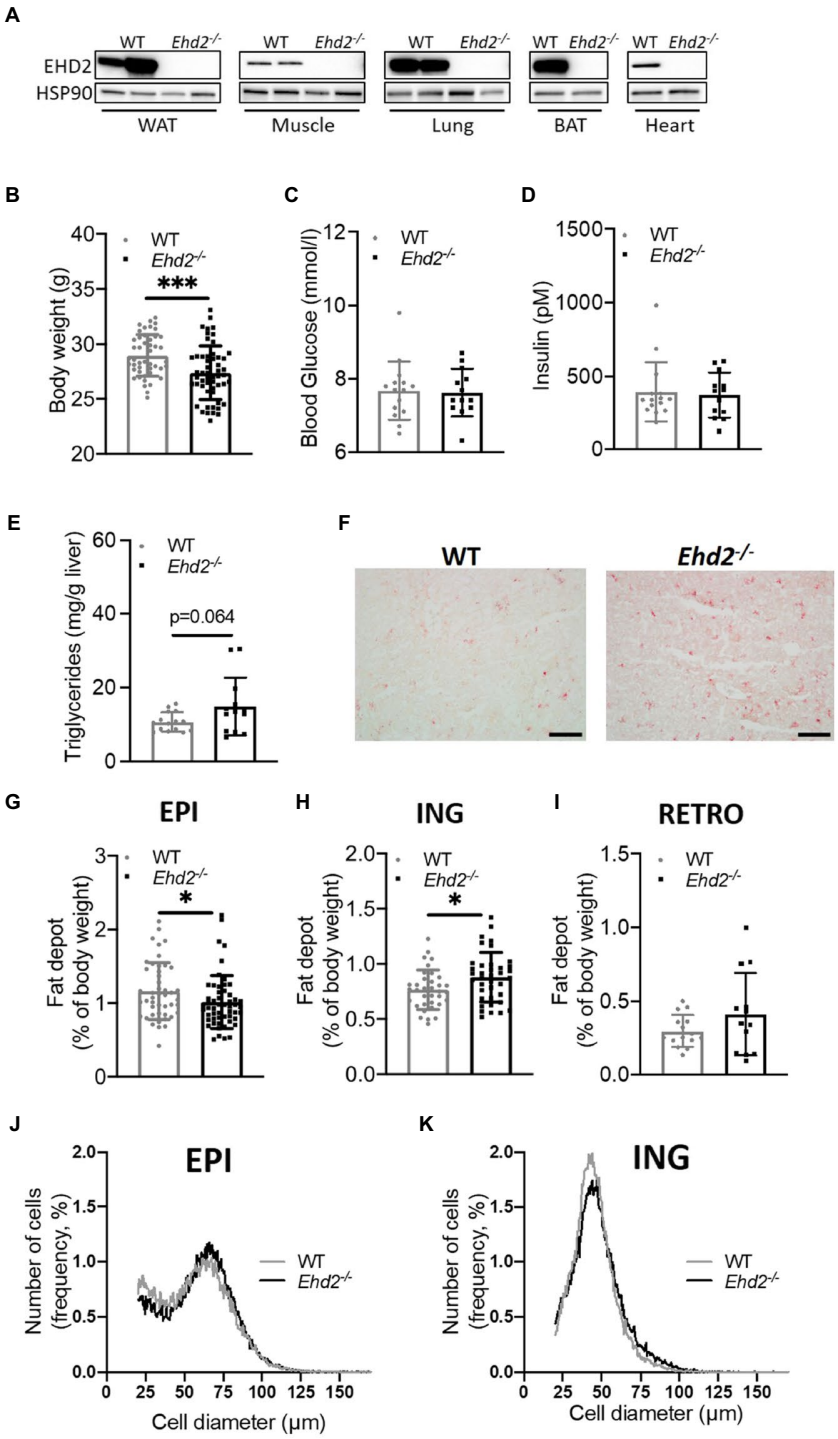
Adipose tissue distribution and adipocyte sizes comparing *Ehd2^−/−^* and WT mice. **(A)** Western blot analysis illustrating expression of EH domain-containing 2 (EHD2) in different tissues; white adipose tissue (WAT), skeletal muscle, lung, brown adipose tissue (BAT), and heart from *Ehd2^−/−^* and WT mice. Comparison of WT and *Ehd2^−/−^* mice fed chow-diet, showing **(B)** body weight (BW; *n*=46–54 animals/group), **(C)** blood glucose (mmol/L; *n*=13–15 animals/group), and **(D)** serum insulin (pM; *n*=13–15 animals/group). **(E)** Liver triglycerides (mg/g liver; *n*=13–15 animals/group), and **(F)** illustrated by Oil-Red-O-staining, scale bar=100μm. Adipose tissue depots weights; **(G)** epididymal (EPI; *n*=46–54 animals/group), **(H)** inguinal (ING; *n*=35–38 animals/group), and **(I)** retroperitoneal (RETRO; 13–15 animals/group). **(J,K)** Adipose cell size distribution of EPI and ING tissue depots analyzed using coulter counter, 6,000 counts/sample, each sample run in duplicate, data displayed as average from *n*=3 samples/group. Data in **(B–E,G–I)** are presented as mean±SD, and ^*^p<0.05 and ^***^p<0.001 were calculated using unpaired two-tailed Student’s *t*-test.

To examine adipocyte function in the EHD2-deficient state, we made use of *Ehd2* whole-body knockout (*Ehd2^−/−^*) mice (Matthaeus et al., 2020). Throughout the study, we used corresponding *Ehd2^+/+^* (WT) mice as control. Western blot analysis from various tissues confirmed global deletion of the EHD2 protein (Figure 1A). Age-matched (~11–12weeks) *Ehd2^−/−^* mice displayed lower body weight (BW), but similar blood glucose and serum insulin levels compared with WT (Figures 1B–D). We found *Ehd2^−/−^* mice to have elevated liver triglyceride levels (*p*=0.064; Figures 1E,F), a decreased amount of EPI adipose tissue, an increased amount of inguinal (ING) adipose tissue, and trends toward an increased amount of retroperitoneal (RETRO) adipose tissue compared with WT (Figures 1G–I). Further, adipocyte size distribution analysis of EPI and ING adipose tissue revealed similar distribution curves comparing *Ehd2^−/−^* and WT mice (Figures 1J,K). These data pinpoint that *Ehd2^−/−^* mice not only have a trend toward increased liver triglycerides, in line with previous report (Claudia Matthäus et al., 2019), but also have reduced visceral fat mass compared with WT.

### Impaired Expansion of Epididymal Adipose Tissue in *Ehd2^−/−^* Mice Following HFD-Feeding

To test if a HFD further aggravated the shifted fat distribution, animals were fed 2weeks of HFD, which has proven sufficient to induce weight gain and systemic insulin resistance in C57Bl6/J mice (Hansson et al., 2018).

**Figure 2:**
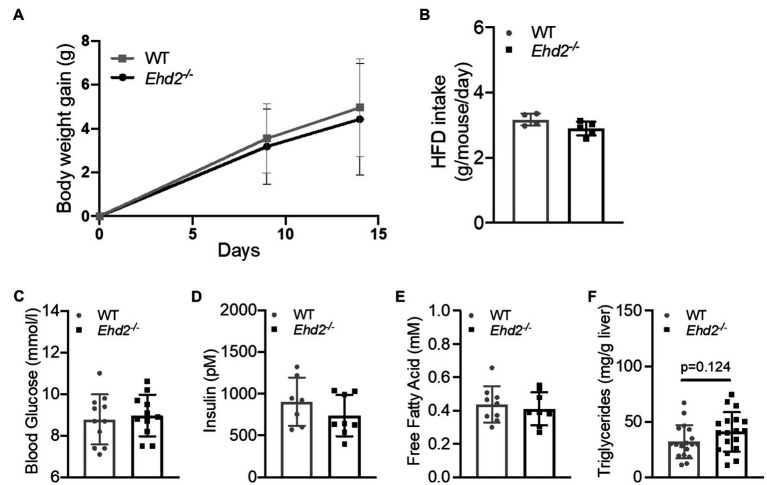
Body weight gain, liver triglycerides, insulin and free fatty acid (FFA) levels in *Ehd2^−/−^* and WT mice following 2weeks of high-fat diet (HFD)-feeding. WT and *Ehd2^−/−^* mice (*n*=18 animals/group) were subjected to HFD-feeding for 2weeks, **(A)** illustrates body weight gain, and **(B)** food intake (gram/mouse/day). **(C)** Final blood glucose (mmol/L; *n*=11), **(D)** serum insulin (pM; *n*=7–8 animals/group), **(E)** serum levels of FFA (mM; *n*=8–9 animals/group), and **(F)** liver triglycerides (mg/g liver; *n*=18 animals/group). Data in **(A–F)** are presented as mean±SD.

**Figure 3:**
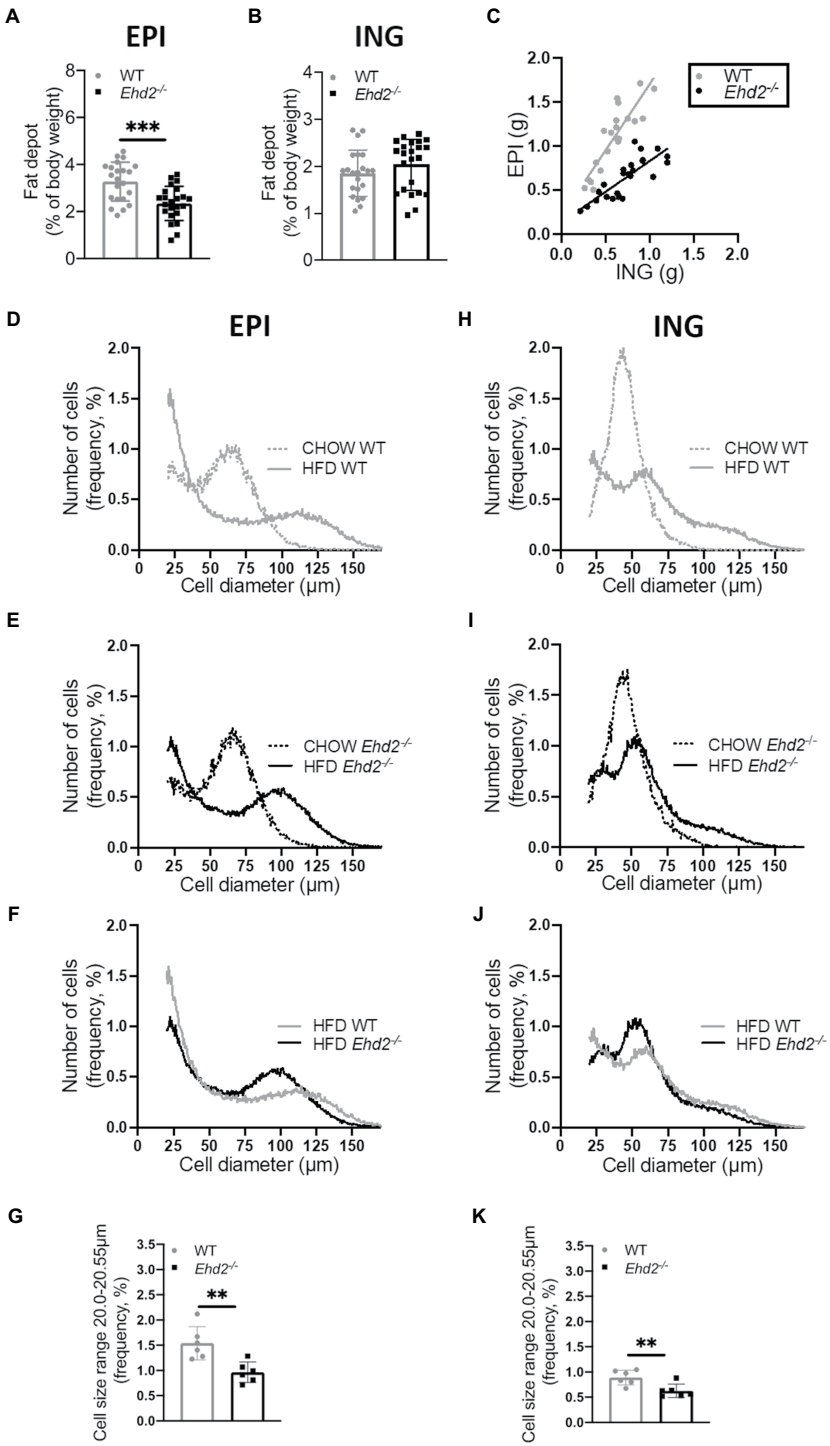
Adipose tissue expansion and adipose cell size distribution in *Ehd2^−/−^* and WT mice following 2weeks of HFD-feeding. **(A,B)** Depot weights of EPI and ING adipose tissue (*n*=22–23 animals/group) collected after 2weeks of HFD. **(C)** Correlation of EPI and ING weight, each symbol (gray=WT, black=*Ehd2^−/−^*) represent one animal, line shows simple linear regression analysis. Adipose cell size distribution obtained by coulter counter in EPI **(D–F)** and ING **(H–J)** depots. Data displayed as average from *n*=3 samples in chow/group, and *n*=6 samples in HFD/group. **(G,K)** Frequency percentage of small cells (cell diameter 20.00–20.55μm) from HFD-fed mice; EPI **(G)** and ING **(K)**, data obtained from cell size distribution curves, shown in **(F,J)**. Data in **(A,B,G,K)** are presented as mean±SD, and ^**^p<0.01 and ^***^p<0.001 were calculated using unpaired two-tailed Student’s *t*-test.

Both *Ehd2^−/−^* and WT mice rapidly gained weight to the same extent (Figure 2A), and no differences in food intake, blood glucose, serum insulin, or serum FFAs levels were observed (Figures 2B–E). Glucose and insulin levels increased significantly, and to the same extent, with HFD-feeding compared with chow in both genotypes (shown in Figures 1C,D, 2C,D), which confirms that C57Bl6/N mice also are prone to rapidly develop obesity and insulin resistance on a high fat diet. There was a trend toward increased liver triglyceride levels in *Ehd2^−/−^* mice compared with WT (Figure 2F). Further, *Ehd2^−/−^* mice fed HFD had a significantly reduced amount of EPI fat tissue, while the ING fat depot was similar with WT (Figures 3A,B). Correlation analysis demonstrated a significant difference in expansion of fat depots, comparing EPI and ING from WT and *Ehd2^−/−^* (Figure 3C), whereas RETRO adipose tissue expansion was similar in *Ehd2^−/−^* compared with WT (Supplementary Figure S1). Cell size distribution analysis revealed a clear shift in adipocyte size distribution toward larger sizes in response to HFD-feeding in both WT and *Ehd2^−/−^* mice [Figures 3D,E (EPI) and Figures 3H,I (ING)]. Even though, mature adipocytes from *Ehd2^−/−^* mice expanded in response to HFD, the distribution curve displayed lower frequency of very small adipocytes and an accumulation of medium-sized adipocytes in *Ehd2^−/−^* compared with WT in the HFD-fed state (Figures 3F,G,J,K).

### Decreased PPARγ Activity and Lowered Expression of Caveolin-1 and FABP4 in Adipocytes From *Ehd2^−/−^* Mice

**Figure 4:**
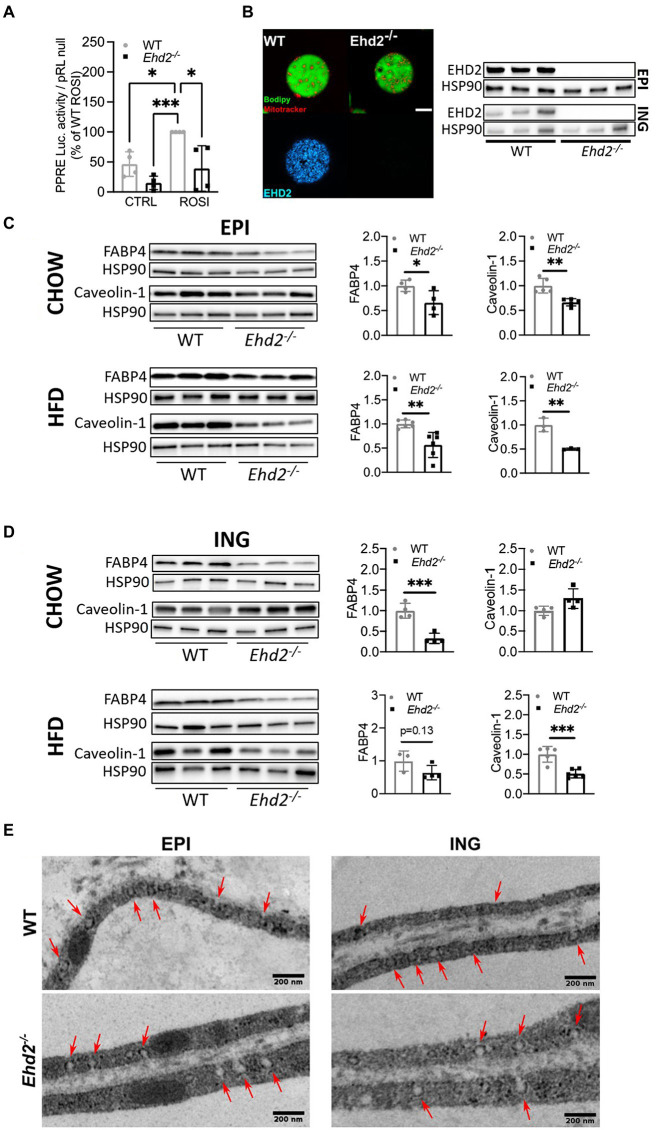
PPARγ activity, FABP4 expression, and caveolin-1 in adipocytes from *Ehd2^−/−^* and WT mice. **(A)** PPARγ activity in primary isolated EPI adipocytes from chow-fed *Ehd2^−/−^* and WT mice, non-stimulated or stimulated with rosiglitazone (ROSI) for 24h using transfection and dual luciferase assay. Data were normalized to Renilla reporter (pRLnull), and expressed as percentage of ROSI WT (*n*=4). **(B)** Left panel: Confocal image of primary EPI adipocytes from WT and *Edh2^−/−^* mice stained for lipids (bodipy, green), mitochondria (Mitotracker, red), and EHD2 (antibody, blue), scale bar=20μm. Right panel: Western blot analysis of EHD2 expression in cell lysates of primary adipocytes isolated from EPI and ING adipose tissue. **(C)** Representative western blots and quantification of total protein levels of FABP4 and caveolin-1 from primary adipocyte lysates from EPI adipose tissue from chow or HFD-fed mice. HSP90 was used as loading control, *n*=3-6/group**. (D)** Same as in **(C)** with lysates from ING adipose tissue. **(E)** Electron micrographs showing the structure and distribution of caveolae at the adipocyte cell membrane in EPI and ING tissue from WT and *Ehd2^−/−^* animals. Arrows show clearly discernible caveolae. Scale bar=200nm. Data in **(A,C,D)** are presented as mean±SD, and ^*^p<0.05, ^**^p<0.01 and ^***^p<0.001 were calculated using one-way ANOVA, Tukey’s *post hoc* test **(A)**, and unpaired two-tailed Student’s *t*-test **(C,D)**.

Ligand-activated transcription factor PPARγ is essential for adipogenesis (Rosen et al., 2002). Since EHD2 has been reported to influence transcriptional activity (Torrino et al., 2018), we next assessed if reduced EPI fat tissue expansion could reflect impaired PPARγ activity in *Ehd2^−/−^* cells by using a PPARγ response element (PPRE) reporter assay. Indeed, primary EPI adipocytes from chow-fed *Ehd2^−/−^* mice displayed 60–70% lower PPARγ activity compared with WT, both in non-stimulated cells, and in cells treated with the PPARγ ligand Rosiglitazone (Figure 4A). Further, by western blot, we detected ~50% lower levels of FABP4 (fatty acid binding protein), one of the target genes of PPARγ (Kletzien et al., 1992), in both EPI and ING adipocytes isolated from *Ehd2^−/−^* mice compared to WT, even though, less pronounced in ING cells following HFD-feeding (Figures 4C,D). The EHD2 knockout was confirmed by western blot using cell lysates and by confocal imaging (Figure 4B). These results suggest that EHD2 influences the transcriptional activity of PPARγ in primary adipocytes, which could explain the impaired expansion of the EPI adipose tissue depot. Possibly, this is due to lowered levels of FABP4 and intracellular fatty acids, the latter acting as endogenous PPARγ ligands, stimulating both lipogenic protein expression and differentiation of new adipocytes. In addition, the impaired response to the synthetic ligand Rosiglitazone in *Ehd2*^−/−^ cells also suggests that PPARγ itself or its co-activators are downregulated.

Further, knock-down of EHD2 is reported to affect the stability of caveolae rather than caveolae density (Moren et al., 2012; Ludwig et al., 2013; Matthaeus et al., 2020). In line with that, we could observe the presence of caveolae in intact adipose tissue from WT and *Ehd2^−^/^−^* by electron microscopy (Figure 4E, arrows indicate caveolae). Western blot analysis revealed that the main core protein of caveolae, caveolin-1, was reduced ~50% in adipocytes isolated from EPI and ING adipose tissue in *Ehd2^−/−^* mice compared to WT after HFD-feeding (Figures 4C,D). Possibly, the lower caveolin-1 expression could affect the composition or stability of caveolar structures.

### Decreased Phosphorylation of Perilipin-1 and HSL, and Decreased Lipolysis in Inguinal Adipocytes From *Ehd2^−/−^* Mice

**Figure 5:**
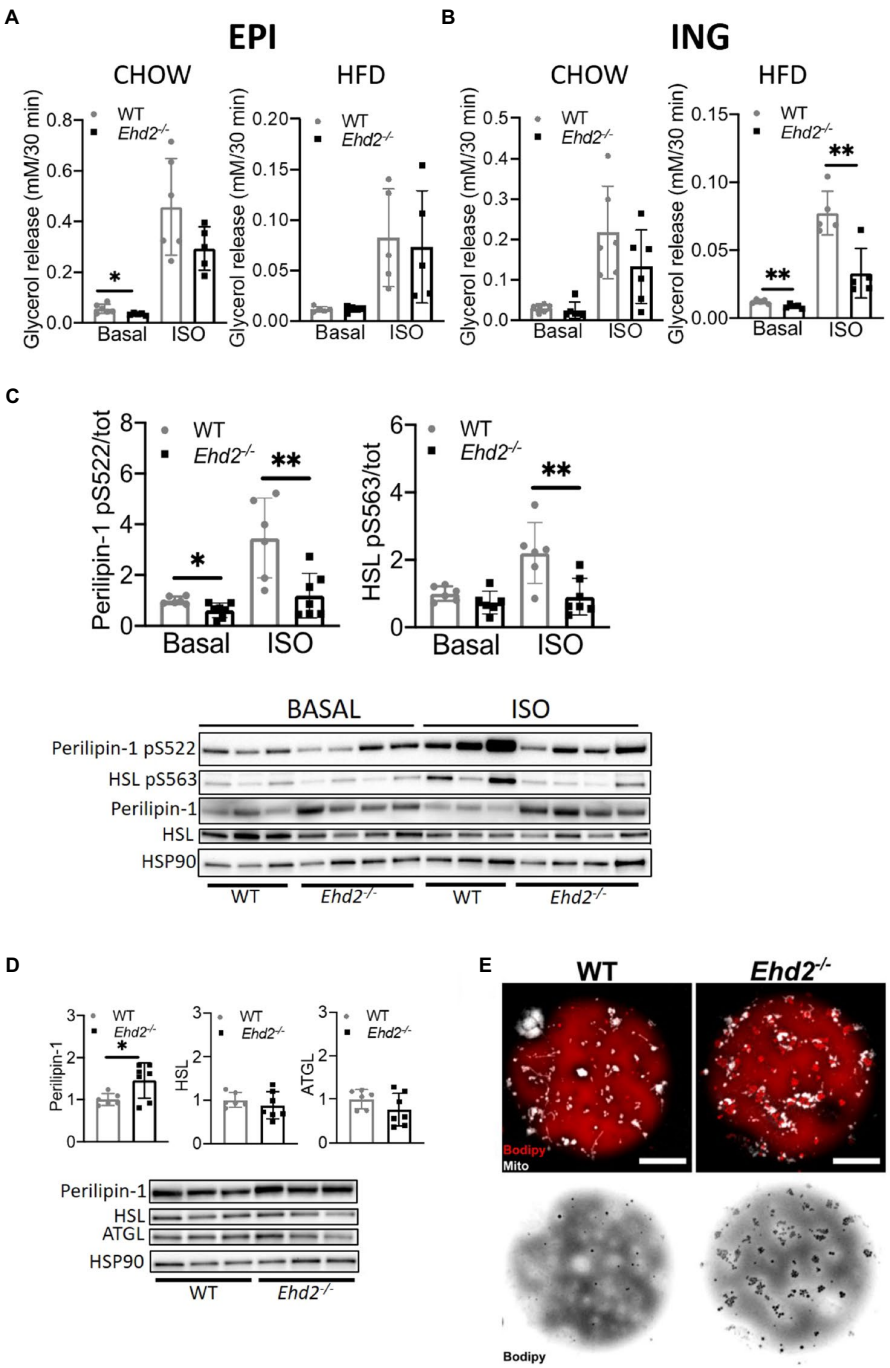
Lipolysis in adipocytes isolated from *Ehd2^−/−^* and WT mice. **(A,B)** Primary adipocytes were isolated from EPI or ING depot, and cells in suspension were subjected to non-stimulation (basal) or β adrenergic stimulation using isoprenaline (ISO; 10nM) for 30min. Lipolysis was measured as release of glycerol into the medium in a 10% (v/v) cell suspension. Basal and ISO stimulated lipolysis of EPI **(A)** and ING **(B)** adipocytes from chow and HFD fed mice (*n*=6 chow, *n*=5 HFD). **(C)** Western blot showing total and phosphorylated levels of perilipin-1 pS522 and HSL pS563 from ING adipocytes from HFD-fed mice, either non-stimulated or stimulated with isoprenaline (ISO; 30min; *n*=6-7/group), quantification shows phosphorylation over total protein. HSP90 used as loading control. **(D)** Western blot analysis displaying representative image and quantification of cell lysates from ING adipocytes after HFD-feeding. Total protein level of perilipin-1, HSL, and adipose triglyceride lipase (ATGL) from non-stimulated cells (*n*=3–4). **(E)** Confocal image of lipid droplet clusters in primary ING adipocytes from chow-fed mice. Cells were stained for lipids (bodipy, red) and mitochondria (mitotracker, white). Lower panel shows only lipids in inverted grayscale. Scale bar=10μm. Data in **(A–D)** are presented as mean±SD, and ^*^p<0.05 and ^**^p<0.01 were calculated using two-tailed Student’s *t*-test.

So far, the data suggest that absence of EHD2 causes impaired expansion of the adipocytes *per se* and also leads to reduced EPI adipose tissue mass. To investigate whether lack of EHD2 affects intracellular lipid handling, we next focused on lipolysis, the hydrolysis of triglycerides. In the following, lipolysis, measured as the release of glycerol in primary adipocytes, was examined in both non-stimulated (basal) and β-adrenergic [Isoprenaline (ISO)] stimulated cells. In chow-fed mice, basal but not ISO-stimulated lipolysis was significantly lower in EPI adipocytes from *Ehd2^−/−^* mice compared with WT (Figure 5A). In contrast, there was no significant difference in lipolysis in EPI adipocytes isolated after HFD-feeding in either non- or ISO stimulated cells comparing WT and *Ehd2^−/−^* (Figure 5A). In ING adipocytes, no significant change was observed in chow-fed mice (Figure 5B). Yet, both basal and ISO stimulated lipolysis was significantly reduced (~50%) in cells obtained from *Ehd2^−/−^* mice compared to WT after HFD-feeding (Figure 5B). To further elucidate mechanisms behind reduced lipolysis in ING cells obtained after HFD-feeding, we analyzed the phosphorylation of perilipin-1 and HSL, the main triglyceride lipase. We found that adipocytes from *Ehd2^−/−^* mice displayed reduced phosphorylation levels of perilipin-1 (pS522) and HSL (pS563) following ISO stimulation (Figure 5C), which is in line with reduced lipolysis (Figure 5B).

Still, the expression of HSL and ATGL was unaltered, whereas total perilipin-1 level was significantly increased (~30%) in *Ehd2^−/−^* adipocytes compared to WT (Figure 5D). Possibly, the latter could reflect an increased lipid droplet surface area since small surface-associated lipid droplets were visible to a larger extent in adipocytes from *Ehd2^−/−^* mice (Figure 5E).

**Figure 6:**
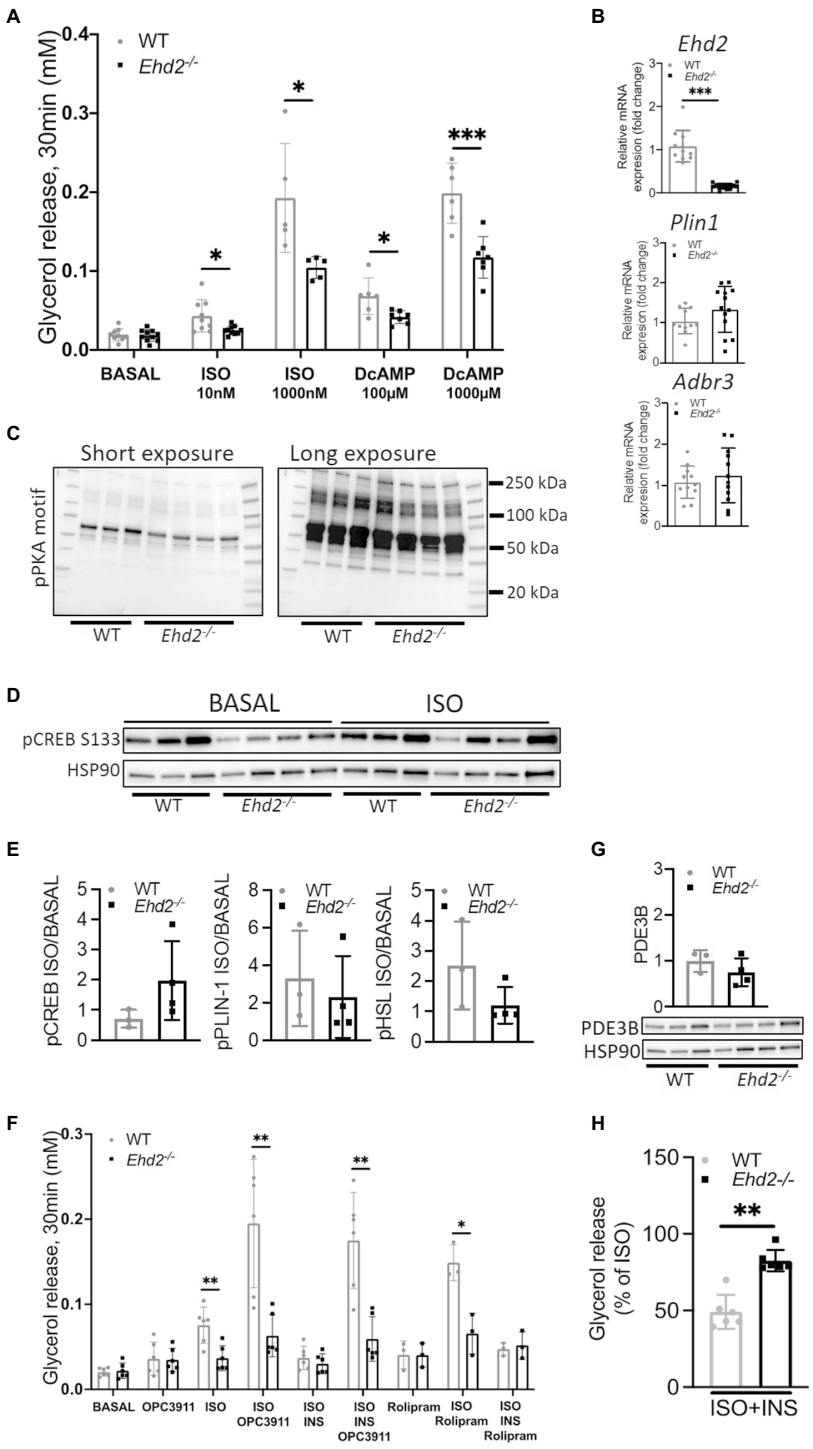
Lipolysis using cAMP analog and PDE-inhibitors in inguinal adipocytes from *Ehd2^−/−^* and WT mice. **(A)** Lipolysis measured as release of glycerol into the medium in a 10% (v/v) cell suspension using primary adipocytes isolated from the ING adipose tissue depot of HFD-fed mice. Cells in suspension were either non-stimulated (basal), or β adrenergic stimulated using isoprenalin (ISO; 10 or 1,000nM), or stimulated with the non-degradable cAMP analogue dibutyryl cAMP (DcAMP; 100 or 1,000μM) for 30min. (*n*=5–8/group). **(B)** mRNA expression of *Plin1*, *Adrb3*, and *Ehd2* from ING adipose tissue of HFD fed mice, assessed by RT-qPCR, 18S rRNA used as reference gene, data plotted as relative fold of WT (*n*=11–13/group). **(C)** Western blot using a phospho protein kinase A (PKA) motif specific antibody [P-RXX(S/T)] in iso-stimulated ING adipocyte lysates from WT (*n*=3) and *Edh2^−/−^* (*n*=4) mice on HFD. **(D)** Western blot showing phosphorylated levels of CREB pS133 in ING adipocytes, either non-stimulated or stimulated with isoprenaline (ISO; 30min), isolated from HFD-fed mice (*n*=3–4/group), HSP90 was used as loading control. **(E)** Quantification of pCREB pS133 [images shown in **(D)**], Perilipin-1 pS522 and HSL pS563 (data re-plotted from Figure 5C), expressed as ISO/BASAL. **(F)** Lipolysis assessed by glycerol release in primary ING adipocytes of HFD-fed mice, where cells in suspension were pre-incubated for 10min with or without PDE3 inhibitor OPC3911 (10μM) or PDE4 inhibitor Rolipram (10μM) followed by 30min incubation in combination with or without isoprenaline (ISO; 10nM) and insulin (1nM). **(G)** Representative image and quantification of total protein levels of PDE3B from primary ING adipocytes from HFD-fed mice (*n*=3–4/group), HSP90 used as loading control. **(H)** Insulin’s (1nM) anti-lipolytic effect in primary ING adipocytes from HFD-fed mice, data extracted from **(F)** and expressed as % of glycerol release from ISO-stimulated cells (*n*=6/group). Data in **(A,B,E–H)** are presented as mean±SD, and ^*^p<0.05, ^**^p<0.01, and ^***^p<0.001 were calculated using two-tailed Student’s *t*-test.

To test if the reduced lipolysis was caused by impaired signaling at the level of beta adrenergic receptor or adenylate cyclase, we treated cells with dibutyryl-cAMP (DcAMP), a cell permeable, non-degradable cAMP analog. DcAMP activates protein kinase A (PKA) which phosphorylates and activates HSL and perilipin-1, and thereby induces lipolysis. Compared to WT, *Ehd2^−/−^* adipocytes showed lower lipolysis upon DcAMP treatment (Figure 6A). This suggests that the impaired lipolysis in *Ehd2^−/−^* adipocytes occurs downstream of cAMP in the beta adrenergic signaling pathway. Notably, in this set of experiments, we could confirm that also at a very high dose of ISO (1,000nM), lipolysis was significantly reduced in *Ehd2^−/−^* adipocytes. Further, these results are also in agreement with similar gene expression of adrenergic receptors (*Adr3b*) in adipocytes from *Ehd2^−/−^* mice compared with WT (Figure 6B). The trends toward increased gene expression of *Plin1* mirrors the increased perilipin-1 protein expression shown in Figure 5D.

Consistent with the impaired effects of ISO and DcAMP on lipolysis and phosphorylation of HSL and perilipin-1, we found reduced ISO-induced phosphorylation of several PKA targets in adipocytes from *Ehd2^−/−^* mice using a phospho-PKA motif specific antibody [P-RXX(S/T); Figure 6C]. Further, we examined the phosphorylation of the cAMP-responsive transcription factor CREB. CREB (pS133) was lower in non-stimulated cells from *Ehd2^−/−^* mice compared with WT but increased in response to ISO (Figure 6D). In comparison, the fold increase of phosphorylated perilipin-1 and HSL in response to ISO was lower in *Ehd2^−/−^* adipocytes, whereas the fold increase of phosphorylated CREB was increased in *Ehd2^−/−^* adipocytes (Figure 6E). Thus, the ability to transmit cAMP-dependent signaling was not compromised for all targets in adipocytes from *Ehd2^−/−^* mice.

### The Anti-lipolytic Effect of Insulin Is Diminished Despite Intact PDE3 Expression and Activity in Adipocytes Isolated From *Ehd2^−/−^* Mice

Next, we investigated if altered expression and activity of PDE3 could explain the impaired lipolysis in ING adipocytes from *Ehd2^−/−^* mice. PDE3B is a major PDE family member expressed in adipocytes, responsible for degradation of cAMP. Thus, the expression level and activity of PDE3B have an impact on cAMP mediated signaling events, particularly the anti-lipolytic effect of insulin (Degerman et al., 2011).

The use of a PDE3 inhibitor (OPC3911) increased basal and ISO-mediated lipolysis to the same extent (~2-fold) in both genotypes (Figure 6F). We also detected similar protein expression of PDE3B in WT and *Ehd2^−/−^* adipocytes (Figure 6G). Still, lower lipolysis in ISO-stimulated cells in *Ehd2^−/−^* adipocytes was observed even in the presence of OPC3911, indicating that increased PDE3B activity is not causing the impaired ISO-mediated lipolysis in *Ehd2^−/−^* adipocytes (Figure 6F).

Further, we investigated insulin’s ability to suppress lipolysis, one of insulin’s major metabolic effects in adipocytes. As expected, insulin efficiently suppressed ISO-induced lipolysis in WT (~50% reduction; Figure 6F, same data normalized to ISO are presented in Figure 6H). In contrast, much less suppression was observed in cells from *Ehd2^−/−^* [~25% reduction (Figures 6F,H)]. Further, when co-stimulating with both OPC3911, ISO, and insulin, the anti-lipolytic effect of insulin was lost in WT as expected, but also in *Ehd2^−/−^* adipocytes (Figure 6F). In comparison, the use of a PDE4 inhibitor, Rolipram, increased ISO-induced lipolysis, but did not abolish insulin’s effect in either genotypes (Figure 6F).

Together, alterations of PDE3B expression/activity appear not to contribute to the low lipolysis observed in adipocytes from *Ehd2^−/−^* mice. Instead, the data suggest that a defect at the level of, or downstream of PKA, contributes to this phenotype.

## Discussion

In the present study, we demonstrate that lack of EHD2 clearly affects intracellular lipid handling, and the overall storage of excess energy. These effects are well in-line with the first description of the *Ehd2*^−/−^ mouse model, in which EHD2 was proposed to regulate fatty acid uptake in a caveolae-dependent fashion, and lowered EHD2 levels in the obese state were suggested to reflect altered lipid handling (Matthaeus et al., 2020).

We previously identified *Ehd2* as one of the highest upregulated genes in adipose tissue during a short-term HFD intervention in mice (Hansson et al., 2018). Later, we confirmed by gene silencing and overexpression *in vitro* that EHD2 was necessary to sustain adipocyte maturation and lipid metabolism (Moren et al., 2019). While the initial characterization of *Ehd2^−/−^* mice reported increased lipid uptake in cultured adipose cell models differentiated *in vitro* (Matthaeus et al., 2020), the novel findings in the current study include a marked impairment in lipolysis in primary adipocytes, and reduced expansion of the epididymal adipose tissue depot in *Ehd2^−/−^* mice.

Based on previous knowledge of how EHD2 influences caveolae stability (Moren et al., 2012; Matthaeus et al., 2020), the EHD2-mediated effect on lipolysis is likely dependent on caveolar stability and/or caveolar property. Caveolin-1, the main structural protein of caveolae, is known to scaffold several signal molecules, among those G-protein coupled receptors (Insel et al., 2005). Moreover, adrenergic receptors were reported to be localized at caveolae (Schwencke et al., 1999), and their downstream signaling components to depend on caveolae integrity (Calaghan et al., 2008). The fact that we found lipolysis to be reduced in adipocytes from *Ehd2^−/−^* also when treated with a cAMP analog (DcAMP), and that the presence of a PDE3-inhibitor did not restore lipolysis, supports that this impairment is to be found downstream of the beta adrenergic receptor and adenylate cyclase. In line with this statement, caveolin-1 was shown to facilitate PKA-mediated phosphorylation of perilipin by bringing PKA physically close enough to fully phosphorylate perilipin in adrenergic-stimulated cells (Cohen et al., 2004). Possibly, the marked reduction of caveolin-1 level in EHD2-deficient cells detected herein could affect spatial PKA orientation, which in turn would affect the transmission of cAMP-dependent signaling downstream of PKA. Further, the increased perilipin-1 level in *Ehd2^−/−^* cells could reflect an increased lipid droplet surface, since we observed an increased number of small surface-associated lipid droplets. The finding that insulin-mediated inhibition of lipolysis was clearly diminished in adipocytes from *Ehd2^−/−^* compared with WT, despite lower adrenergic stimulated lipolysis, suggests that not only the response to beta adrenergic stimulation but also the response to insulin is hampered in *Ehd2^−/−^* cells, which definitely is of interest to follow up in future work. Together, we propose that EHD2 is required to sustain signaling events relying on caveolae and interaction with its integral components.

The marked impairment of epididymal adipose tissue expansion in *Ehd2^−/−^* mice could be caused by impaired adipogenesis, and an inability to increase the adipocyte number and pool of small cells (Faust et al., 1978). In support of this, we found lower proportion of small adipocytes, and that EPI adipocytes had attenuated PPARγ activity, the key driver of adipocyte differentiation (Rosen et al., 2002). Possibly, in adipocytes, EHD2 influences gene expression indirectly by affecting flux of intracellular fatty acids, which serve as PPARγ ligands. This is well in-line with previous reports, where EHD2 was proposed to exert transcriptional control (Torrino et al., 2018). Unfortunately, due to technical limitations, we could not verify PPARγ activity in isolated adipocytes from the inguinal depot. Thus, while primary adipocytes *per se* could expand despite EHD2 deficiency, the deposit of excess energy was, at the whole-body level, clearly shifted. Indeed, following HFD-feeding, adipogenesis occurs primarily in epididymal and not in inguinal adipose tissue depot (Wang et al., 2013; Jeffery et al., 2015), supporting the hypothesis that differences in depot weight following HFD-feeding in *Ehd2^−/−^* mice reflect a reduced capacity to promote differentiation.

In contrast, the inguinal adipose tissue depot expanded similar comparing the two genotypes, but still displayed a similar decrease in small adipocytes and increase in medium-sized adipocytes as observed in the epididymal adipose tissue depot. Therefore, the impaired lipolysis in ING adipocytes cannot be explained by an increased pool of hypertrophic cells (Laurencikiene et al., 2011). Rather, depot-specific effects related to caveolar stability or function could explain this, and need to be addressed in future studies. The fact that the most prominent impairment of lipolysis was found in the beta adrenergically stimulated condition, or in experiments, where lipolysis was pharmacologically activated using a cAMP analog, could explain why the inguinal fat mass was not increased further in *Ehd2^−/−^* compared with WT. *Ehd2^−/−^* mice’s ability to expand the subcutaneous adipose tissue could at least in part explain that similar levels of FFA, insulin, and glucose were observed in *Ehd2^−/−^* compared with WT mice, despite slightly elevated liver triglyceride levels.

Taken together, in the present study, we report severe impairments of adipogenesis in the epididymal depot specifically, in EHD2 deficient mice. We also report that EHD2 is an essential component to sustain beta adrenergic lipolysis in inguinal adipocytes, possibly indirectly by influencing caveolae stability and thereby their ability to orchestrate signaling events. Thus, the discovery of EHD2 as an important factor involved in lipid handling in specific adipose tissue depots could provide important insights, necessary to detangle the mechanisms that ultimately limits the cell’s ability to store excess energy.

## Data Availability Statement

The raw data supporting the conclusions of this article will be made available by the authors, without undue reservation.

## Ethics Statement

The animal study was reviewed and approved by Malmö/Lund Committee for Animal Experiment Ethics, Lund, Sweden.

## Author Contributions

KS, CF, ED, and BM conceived and designed the experiments. KS, CF, BM, CM, SS, and MG collected, analyzed, and interpreted the data. KS, CF, BM, CM, and ED drafted the article or revised it critically for important intellectual content. All authors made comments on the manuscript and approved the final version submitted for publication.

## Funding

This work was financially supported by the Swedish Research Council (2019-00978), Strategic Research Area Exodiab (2009-1039), the Swedish Foundation for Strategic Research (IRC15-0067), Novo Nordisk (NNF17OC0027054 and NNF20OC0063659), Swedish Diabetes Foundation, The Crafoord Foundation, Albert Påhlsson Foundation, Hjelt Foundation, and the Royal Physiographic Society in Lund.

## Conflict of Interest

The authors declare that the research was conducted in the absence of any commercial or financial relationships that could be construed as a potential conflict of interest.

## Publisher’s Note

All claims expressed in this article are solely those of the authors and do not necessarily represent those of their affiliated organizations, or those of the publisher, the editors and the reviewers. Any product that may be evaluated in this article, or claim that may be made by its manufacturer, is not guaranteed or endorsed by the publisher.

## Supplementary Material

Supplementary Figure S1 Expansion of retroperitoneal (RETRO) fat depot following 2weeks of HFD. Depot weight of RETRO adipose tissue (*n*=22 animals/group) collected from HFD-fed mice. Correlation of RETRO weight and body weight gain. Correlation of RETRO weight and epididymal or inguinal weight, each symbol (gray=WT; black=*Ehd2^−/−^*) represent one animal, line shows simple linear regression analysis.
