# Supplementary figures and images for: EH Domain-Containing 2 Deficiency Restricts Adipose Tissue Expansion and Impairs Lipolysis in Primary Inguinal Adipocytes
Source: Front Physiol. 2021 Sep 24;12:740666. doi: 10.3389/fphys.2021.740666 (PMC8497890; doi:10.3389/fphys.2021.740666)

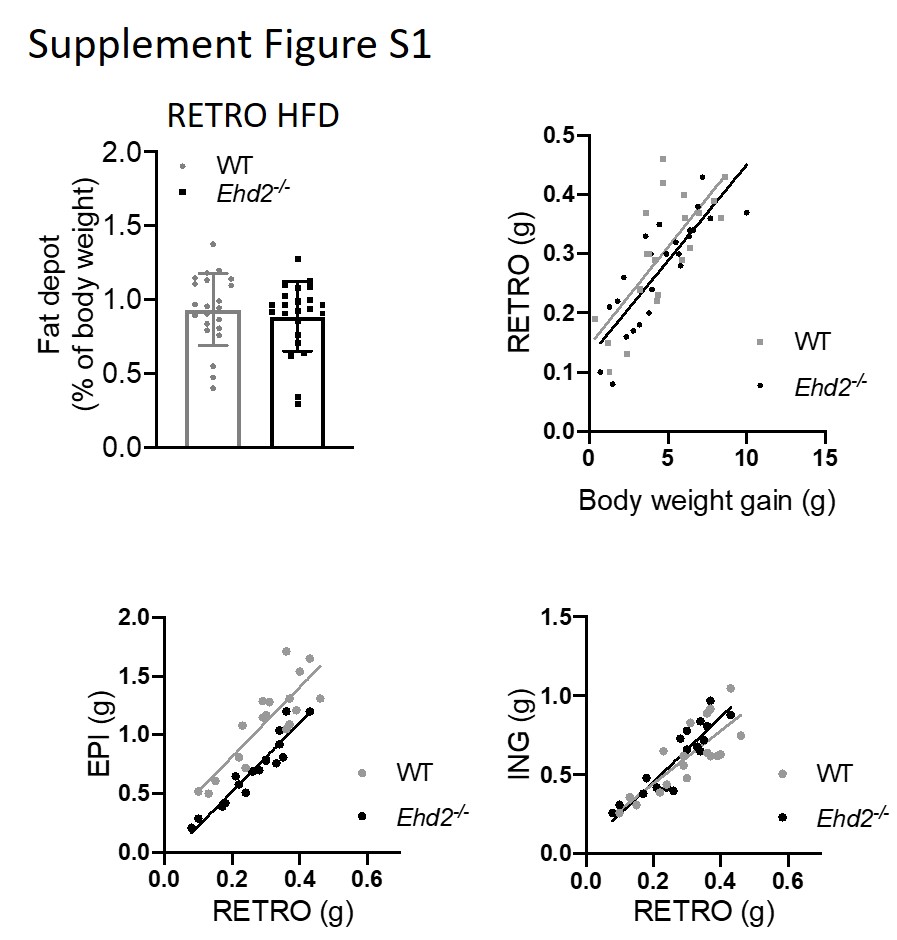

Supplement: Supplementary Figure S1 — Expansion of retroperitoneal (RETRO) fat depot following 2weeks of HFD. Depot weight of RETRO adipose tissue (n=22 animals/group) collected from HFD-fed mice. Correlation of RETRO weight and body weight gain. Correlation of RETRO weight and epididymal or inguinal weight, each symbol (gray=WT; black=Ehd2−/−) represent one animal, line shows simple linear regression analysis. [file Image_1.JPEG]
